# Draft Genome Sequence of the Fungus *Paraphoma* sp. B47-9, a Producer of a Biodegradable Plastic–Degrading Enzyme

**DOI:** 10.1128/genomeA.01159-16

**Published:** 2016-10-20

**Authors:** Yuka Sameshima-Yamashita, Hideaki Koike, Motoo Koitabashi, Azusa Saika, Tomotake Morita, Tohru Yarimizu, Hiroko Kitamoto

**Affiliations:** a National Agriculture and Food Research Organization (NARO), Tsukuba, Ibaraki, Japan; b Research Fellow of the Japan Society for the Promotion of Science, Chiyoda-ku, Tokyo, Japan; c National Institute of Advanced Industrial Science and Technology (AIST), Tsukuba, Ibaraki, Japan

## Abstract

*Paraphoma* sp. B47-9 is a producer of a biodegradable plastic–degrading enzyme. Here, we report the draft genome sequence of this strain. The draft genome assembly has a size of 39.3 Mb with a GC content of 52.4% and consists of 185 scaffolds.

## GENOME ANNOUNCEMENT

Fungal strains closely related to *Paraphoma* have the ability to degrade biodegradable plastic (BP) films (1). The best degrader, *Paraphoma* sp. B47-9 (1), isolated from the leaf surface of barley in May 2006 in Tsukuba, Ibaraki, Japan, secretes a cutinase-like BP-degrading enzyme (PCLE) (2–4). Spray treatment of the culture filtrate with PCLE significantly accelerated the degradation of BP agricultural mulch films in the field (5). Thus, this treatment is promising for improving the efficiency of plowing down used BP films in the field using farm equipment. Here, we present the draft genome sequence of strain B47-9. The availability of this genome sequence should enable researchers to improve the productivity of BP-degrading enzymes. 

A paired-end DNA library (insert size: ~500 bp) of genomic DNA of B47-9 was prepared using a NEBNext Ultra DNA library prep kit for Illumina (New England BioLabs, Ipswich, MA, USA) and was sequenced on the Illumina MiSeq system. A total of 6.94 Mb paired-end reads, each 250 bp in length, were generated. In addition, a mate-paired library (insert size: ~3,000 bp) was prepared using a Nextera mate-pair sample prep kit (Illumina, San Diego, CA, USA). This library was sequenced and resulted in 10.0 Mb mate-paired reads. The Allpath-LG assembler (6) was used to generate 576 contigs with 37.8× and 56.5× genome coverages from the paired-end and mate-paired libraries, respectively. The contigs derived from the two libraries were assembled into 185 scaffolds. Accordingly, the draft genome size of fungal strain B47-9 was estimated to be 39.3 Mb with a 52.4% GC content. The length of the longest scaffold was 2.4 Mb, and the *N*_50_ length was 1.1 Mb with 12 scaffolds.

Using the database of *Aspergillus nidulans* FGSC A4 as a reference, 14,202 protein-coding genes were automatically predicted using the AUGUSTUS program (7). Among them, 12,094 genes were homologous to proteins in the RefSeq database (release 65).

PCLE belongs to the cutinase (EC3.1.1.74) family. Draft genome sequencing revealed six putative cutinase genes with the cutinase motif sequence. Among them, the gene responsible for PCLE was located at scaffold 53. Although *Paraphoma* sp. B47-9 is not pathogenic to plants, a BLAST search revealed that the amino acid sequence of PCLE showed the highest identity (65%) and positivity (75%) with one of the cutinases (SNOG_16132) in *Stagonospora nodorum*, a major necrotrophic fungal pathogen of wheat (8), and 54 to 58% identity and 73 to 76% positivity with three cutinases (CUT1, CUT2, CUT3) of *Fusarium solani* f. sp. *pisi*, which is virulent against pea (9). Within the B47-9 genomic DNA, there are also homologues of genes related to the transcription of cutinases in *F. solani* (9). These findings suggest that the draft genome sequence should promote our understanding of the mechanism of expression of PCLE in B47-9.

### Accession number(s).

The nucleotide sequences of the *Paraphoma* sp. B47-9 genome have been deposited in DDBJ/EMBL/GenBank under the accession numbers BCLK01000001 to BCLK01000576.
